# Evaluation of the efficacy of meloxicam for post-operative management of pain and inflammation in horses after orthopaedic surgery in a placebo controlled clinical field trial

**DOI:** 10.1186/s12917-015-0427-4

**Published:** 2015-05-15

**Authors:** Ulrich Walliser, Albrecht Fenner, Nicole Mohren, Thomas Keefe, Frerich deVries, Chris Rundfeldt

**Affiliations:** Clinic for Horses Kirchheim, Nuertingerstrasse 200, 73230 Kirchheim, Germany; Boehringer Ingelheim Vetmedica GmbH, 55216 Ingelheim am Rhein, Germany; Department of Environmental & Radiological Health Sciences, College of Veterinary Medicine & Biomedical Sciences, Colorado State University, Fort Collins, USA; Drug-Consulting Network, 01445 Coswig, Germany

**Keywords:** Horse, Orthopaedic surgery, Post-operative, Pain control, Non-steroidal anti-inflammatory, Meloxicam, Splint bone fracture, Partial resection, Lameness at trot

## Abstract

**Background:**

The benefit of pre and post-operative administration of non-steroidal anti-inflammatory drugs for the relief of post-operative pain and control of inflammation in horses following orthopaedic surgery has not been previously investigated in controlled clinical field trials, and the utility of such treatment is a matter of ongoing dispute. Recently the utility of post-operative pain management was emphasized. It was therefore our aim to determine the efficacy of meloxicam in horses following partial resection of fractured splint bones. This condition was selected since the limited extent of the insult and the defined surgical intervention allowed the conduct of a randomized, double blinded, placebo-controlled, parallel group, multi-centre clinical field study in a homogenous patient population.

**Results:**

Sixty-six client owned horses requiring unilateral partial splint bone resection were recruited in 15 centres in Germany and were allocated in a 1:1 ratio to receive meloxicam, 0.6 mg/kg for 5 days.

Lameness at trot grades prior to surgery were similar in the meloxicam and placebo treatment groups but were significantly lower in the meloxicam group on day 6 post surgery. Clinical scores for soft tissue swelling and assessment of analgesic and anti-inflammatory efficacy by the investigators at the end of the study were significantly better for the meloxicam compared to the placebo group. No treatment-related adverse reactions were observed.

**Conclusion:**

The administration of meloxicam i.v. once prior to surgery followed by once daily oral administration for four consecutive days is efficacious for the control of post-operative pain and inflammation in horses undergoing orthopaedic surgery.

## Background

Surgical intervention on distal extremities in horses is a standard of care for many orthopaedic conditions including splint bone fractures, condylar fractures, arthroscopic joint revisions, and various other conditions [1-3]. While orthopaedic surgical procedures are well established, the medical management of pain and inflammation resulting from such intervention remains to be an under-addressed field. The benefit of pain management during and after orthopaedic surgery is a matter of controversial discussion. In the past it has been suggested that analgesics be withheld in equine patients to maintain protective reflexes and reduce the risk of injury [4], but more recently the utility of post-operative pain management was emphasized [4-6].

Nonsteroidal anti-inflammatory drugs (NSAIDs) are extensively used as analgesics in veterinary medicine, having proven efficacy in dogs and cats for post-operative pain management in orthopaedic surgery [7]. NSAIDs combine analgesia with anti-inflammatory activity. This pharmacology is the result of their inhibition of the cyclooxygenase pathway and prevention of the formation of pro-inflammatory prostaglandins at the site of injury [8]. Despite recommendations for the use of NSAIDs in equine orthopaedic surgery [5,9,10], evidence for efficacy from controlled clinical field studies is limited [4,11]. Meloxicam is an NSAID which is licensed and widely used for the treatment of pre- and post-operative pain and inflammation following orthopaedic and soft tissue surgery in cats and dogs [12]. It is licensed for use in horses for the alleviation of inflammation and relief of pain in both acute and chronic musculo-skeletal disorders as well as for the relief of pain associated with colic [13]. The anti-inflammatory efficacy of meloxicam in equines has been demonstrated in pharmacodynamic and pharmacokinetic studies [14,15]. But to date no data are available supporting its use in post-operative pain control in orthopaedic surgery.

The objective of this study was to examine the efficacy of meloxicam for the control of post-operative pain and inflammation in horses undergoing orthopaedic surgery in a double-blind placebo-controlled study. Partial resection of fractured splint bones is a small, well defined surgical intervention. This condition was therefore selected as a condition to demonstrate clinical efficacy of meloxicam. Both, the limited extent of pre-surgical pain and the limited and standardized surgical intervention, which involves only non-weight bearing structures and which likely does not result in severe pain, enables a placebo controlled study approach. Part of the data have been previously published in abstract form [16].

## Methods

The study was designed as a randomized, double blind, placebo-controlled parallel group multi-centre clinical field study using two treatment groups with equal numbers of clinical cases per group and was conducted in compliance with good clinical practice [17]. The use of a placebo in the clinical field trial setting of the present study can be justified by the low level of post-operative pain induced by the limited surgical intervention, non-availability of a licensed veterinary medicine for perioperative treatment, or at least a drug with proven clinical evidence for efficacy, as comparator for the clinical indication investigated. The study was approved by the local competent authority of the principle investigator Dr. Walliser, Regierungspräsidium Karlsruhe, Baden-Würtemberg, Az. 35–9182.00, and by all local authorities of all participating study centres in full compliance with German drug law, and by the local animal welfare officer of Boehringer Ingelheim Vetmedica GmbH.

### Animals

Client owned horses requiring orthopaedic surgery for a closed splint bone fracture associated with marked exostosis and development of connective tissue indurations were eligible for inclusion in the study with the consent of the owner. Exclusion criteria were defined as pregnant mares, foals less than 6 weeks of age, horses with distal fractures of the splint bones with absolutely non-reactive tissue, compound fractures, proximal fractures which required internal fixation, lameness of other origin (e.g. coffin joint, fetlock joint), or clinical chemistry values indicative of hepatic, renal or hemorrhagic disease. Prior treatment with short acting corticosteroids or NSAIDs during the previous 8 days or treatment with long acting corticosteroids during the previous 8 weeks also precluded inclusion in the study.

### Treatments

Premedication for surgery was performed according to the study site’s usual practices (except for any treatment that might have affected or masked the clinical symptoms of post-operative pain (e.g. NSAIDs and corticosteroids). For pain control during surgery only drugs with duration of action of up to 4 hours were permitted. In most cases L-methadone and/or ketamine were administered; the use of these analgesic agents did not differ between the two treatment groups. Induction and maintenance of inhalation anaesthesia was also performed according to the study site’s usual practices. To control for potential exaggerated pain after surgery during the study conduct, butorphanol was allowed as a rescue medication for the control of very strong pain but was not required or used in any case.

After an initial examination and confirmation of eligibility for inclusion in the study, the horses were allocated according to a randomization list to one of two treatments as follows: (i) a single intravenous injection of meloxicam^a^ (0.6 mg/kg b.w.) immediately prior to premedication followed by (starting on the following morning) 4 once daily oral administrations of meloxicam^b^ at the same dosage; or (ii) a single intravenous injection of placebo immediately prior to premedication followed (starting on the following morning) by 4 once daily equivalent volume oral administrations of placebo. The parenteral and oral investigational interventions for the two treatment groups looked identical and were identically packaged and labelled. The formulation of the placebo was identical to that of the active treatment except for the absence of the active ingredient. The administration of the interventions was carried out by the blinded investigator at each study site.

### Clinical examinations

General characteristics (e.g. age, breed, and bodyweight), blood chemistry profile, affected limb, site and date of the splint bone fracture, and duration of lameness before surgery were recorded, and diagnosis was confirmed by radiographic assessment before surgery. Daily monitoring included body temperature (°C), heart rate (beats/minute), respiratory rate (breaths/minute) and food intake (scoring system: 0, unchanged (normal); 1, reduced by ≤ 1/3; 2, by ≥ 1/3; 3, none). Immediate post-surgery recovery was assessed in terms of the number of attempts which the horses made to stand until they actually managed to remain standing upright and the interval between the time of extubation and the time when the horse was able to remain standing upright.

The primary endpoint was prospectively defined as lameness at trot (LAMET) (prior to and at 3 and 6 days after surgery) according to the severity of lameness grading system of the American Association of Equine Practitioners (AAEP) [18]. LAMET was not assessed on day 1 post surgery due to the proximity to the surgery. To evaluate the level of pain perception at rest throughout the study, lameness at walk (LAMEW) was assessed prior to and at 1, 3 and 6 days after surgery. Assessment of soft tissue swelling, wound healing and analgesic and anti-inflammatory efficacy were additional secondary endpoints. At the surgical site, soft tissue swelling was evaluated on Days 1, 3 and 6, and local palpatory pain on Days 3 and 6, using a 4 point scoring system for both endpoints with 0, 1, 2, 3 indicating none, mild, moderate and severe, respectively. The circumference (cm) of the affected area and wound healing at the surgical site were evaluated on Days 3 and 6; wound healing was scored as either: 0, no signs of complications; 1, increased local temperature; 2, wound secretion; 3, suture dehiscence and 4, other complications such as wound infections. Dressings were changed on Days 3 and 6. At the end of the study the investigator provided a general assessment of analgesic and anti-inflammatory efficacy of the treatment for each horse using a 4 point grading system whereby 1, 2, 3 and 4 denoted excellent, good, moderate and poor, respectively.

### Data analyses

Baseline characteristics for the horses in the study were summarized using descriptive statistics. Data for LAMET and LAMEW were analysed using ordinal logistic regression (OLR) analysis [19] with treatment as the main effect, first with all six AAEP categories and then for LAMET also with a reduced lameness scale, which was constructed post-hoc to focus on clinically relevant lameness at trot (CRLAT): 1, none or minimal (i.e. combination of AAEP grades 0 and 1); 2, mild (i.e. AAEP grade 2); and 3, moderate to severe (i.e. combination of AAEP grades 3, 4 and 5). To adjust for potential differences in severity of lameness prior to treatment (Day 0), the LAMET and CRLAT data on both study days 3 and 6 were also evaluated using the OLR analysis with adjustment for the respective values at Day 0. For this purpose, the OLR model included two categorical variables, treatment (Meloxicam or placebo) as the main effect and LAMET or CRLAT, respectively, at Day 0 as a potential confounder [19,20].

Data on the categorical secondary efficacy variables (assessments of soft tissue swelling and local palpatory pain at the surgery site, wound healing and the general assessment of analgesic and anti-inflammatory efficacy) were evaluated statistically via chi-square analyses. Data on the quantitative traits, i.e. circumference of the most severely affected area, rectal temperature, heart rate, and respiratory rate, were evaluated using repeated-measures analysis of variance or covariance with treatment and day as fixed effects and, for each of the three physiological variables, with the corresponding values at Day 0 as a covariate. The analysis of each of these four variables included Tukey-Kramer mean comparisons between the meloxicam and placebo treatment groups both overall and on each study day. All statistical analyses were carried out using the SAS computer software package [20].

## Results

A total of 15 investigators contributed 66 clinical cases in the study. Six of the cases enrolled (two in meloxicam and four in placebo group) were subsequently found to be in violation of the inclusion/exclusion criteria and were not further considered in the evaluation of the results for efficacy. Two cases on placebo had two fractures; one horse on placebo got a 2^nd^ fracture on the day after surgery preventing adequate evaluation of lameness compared to pre-surgery; in three horses (one in the placebo group and two in the meloxicam group), the required pre-study procedures were not performed according to protocol or were not documented correctly, preventing the evaluation of treatment efficacy for these cases. All 6 were excluded from the primary study population.

Population characteristics of the meloxicam and placebo groups were well balanced. In particular, more than 80 % of the horses in both treatment groups were Warmbloods, and the two treatment groups were well balanced with respect to sex, age, bodyweight and duration of lameness prior to surgery (Table 1). The treatment groups were broadly similar with regard to the radiographic assessment of the affected splint bone prior to surgery and with regard to both the location of the fracture and the severity of callus formation. Fractures were most frequently presented in one of the forelimbs in both treatment groups and represented 54.8 and 69.0 % of the cases in the meloxicam and placebo groups, respectively. The distribution of fractures between left and right limbs was equal for the two groups. Mild to moderate callus formation was observed in 96.8 and 93.1 % of the cases in the meloxicam and placebo groups, respectively.

**Table 1:** Demographics of study population

Treatment	Number of cases [n]	Race distribution: Warmblood /Pony / Other [%]	Sex: Female /Male / Neutered male [%]	Age [years, mean ± SD]	Weight [kg, mean ± SD]	Lameness duration [days, mean ± SD]
Meloxicam	31	83.3 / 13.3 / 3.3	60 / 4 / 36	9.5 ± 4.9	537 ± 96	23.2 ± 18.4
Placebo	29	86.2 / 3.5 / 10.4	52.2 / 13 / 34.8	10.2 ± 3.7	539 ± 107	25.0 ± 24.8

Lameness at trot (LAMET) was determined on Day 0 prior to surgery and again on Day 3 and Day 6; the distribution of LAMET scores are summarised in Table 2. As determined by OLR, the lameness scores on Day 6 were significantly lower in the meloxicam group as compared to the placebo group (p = 0.007), indicating a significant clinical effect of meloxicam treatment. A similar trend towards a positive treatment effect, although not statistically significant (p = 0.067), was seen on Day 3. However, more cases with no or minimal lameness were included in the meloxicam group; 38.8 % of horses in the meloxicam group had either no or only minimal lameness at trot on Day 0 compared to 20.6 % for the placebo group (Table 1). The frequency of horses with moderate lameness (grade 3) was comparable in both groups (48.4 % and 55.2 %). While the difference in LAMET on Day 0 was not statistically significant (p = 0.162), a baseline adjustment in the evaluation of the treatment success was deemed appropriate to correct for a potential advantage for the meloxicam group. Even with baseline adjustment, the meloxicam-treated animals had significantly less severe lameness on Day 6 (p = 0.040, Table 3). Application of the same statistical analyses to the data on the more clinically relevant lameness scale CRLAT yielded essentially similar results, however the treatment effect on Day 3 was now very close to being significant (p = 0.053, Table 3).

**Table 2:** Distribution of study horses by the AAEP grade for lameness at trot (LAMET) on study days 0, 3, and 6 for the meloxicam and placebo treatment groups

Lameness at Trot		Meloxicam		Placebo	
Day	Grade	N	%	N	%
**Day 0**	None (0)	10	32.3	3	10.3
	Minimal (1)	2	6.5	3	10.3
	Mild (2)	3	9.7	5	17.2
	Moderate (3)	15	48.4	16	55.2
	Serious (4)	1	3.2	2	6.9
	Severe (5)	0	0.0	0	0.0
**Day 3**	None (0)	8	25.8	4	13.8
	Minimal (1)	3	9.7	2	6.9
	Mild (2)	8	25.8	4	13.8
	Moderate (3)	9	29.0	15	51.7
	Serious (4)	3	9.7	3	10.3
	Severe (5)	0	0.0	1	3.5
**Day 6**	None (0)	12	38.7	4	13.8
	Minimal (1)	11	35.5	11	37.9
	Mild (2)	5	16.1	2	6.9
	Moderate (3)	3	9.7	10	34.5
	Serious (4)	0	0.0	2	6.9
	Severe (5)	0	0.0	0	0.0
	**Total**	**31**	**100.0**	**29**	**100.0**

**Table 3:** Summary of the statistical analysis of the primary parameter lameness at trot (LAMET) and clinically relevant lameness at trot (CRLAT)

Variables	Probability value	Variables	Probability value
**LAMET on Day:**		**CRLAT on Day:**	
Day 0	0.162	Day 0	0.256
Day 3	0.067	Day 3	**0.053**
Day 6	**0.007**	Day 6	**0.027**
**LAMET adjusted for day 0 value at Day:**		**CLRAT adjusted for day 0 value at Day:**	
Day 3	0.479	Day 3	0.112
Day 6	**0.040**	Day 6	**0.043**

Results of the ordinal logistic regression (OLR) analysis of LAMET and CRLAT at Days 0, 3, and 6 and at Days 3 and 6, adjusted for the values at Day 0, based on including Day 0 in the OLR model as a categorical variable. CRLAT calculation was based on combining AEEP grades 0 and 1 and combining grades 3 to 5 to generate a 3-point scale.

LAMEW scores were very low prior to, on the day after surgery, and throughout the study (Table 4). In fact, the measurements taken indicated only scores of 0 to 2 (none, minimal, or mild lameness), and the majority of measurements throughout the study reflected a score of 0, representing no lameness at all at walk. There was no difference between the placebo and the treatment group for this measure at any day, and the post-surgical grades were not significantly higher than the pre-surgical ones. There was only one animal from the placebo group which was reported on day 3 only to have a LAMEW score exceeding 2. The animal otherwise did not show any clinically relevant sign of pain at rest and therefore rescue medication was not applied.

**Table 4:** Distribution of study horses by the AAEP grade for lameness at walk (LAMEW) on study days 0, 1, 3, and 6 for the meloxicam and placebo treatment groups

Lameness at Walk		Meloxicam		Placebo	
Day	Grade	N	%	N	%
**Day 0**	None (0)	22	71.0	21	72.4
	Minimal (1)	1	3.2	4	13.8
	Mild (2)	8	25.8	4	13.8
	Moderate to severe (3–5)^1^	0	0.0	0	0.0
**Day 1**	None (0)	20	64.5	16	55.2
	Minimal (1)	2	6.5	6	20.7
	Mild (2)	9	29.0	7	24.1
	Moderate to severe (3–5)^1^	0	0.0	0	0.0
**Day 3**	None (0)	22	71.0	19	65.5
	Minimal (1)	7	22.6	6	20.7
	Mild (2)	2	6.5	3	10.3
	Moderate to severe (3–5)^1^	0	0.0	1	3.4
**Day 6**	None (0)	29	93.5	23	79.3
	Minimal (1)	2	6.5	4	13.8
	Mild (2)	0	0.0	2	6.9
	Moderate to severe (3–5)^1^	0	0.0	0	0.0
	**Total**	**31**	**100.0**	**29**	**100.0**

^1^Since all but one horse had LAMEW grades lower than 3, the grades 3–5 were combined.

**Figure 1:**
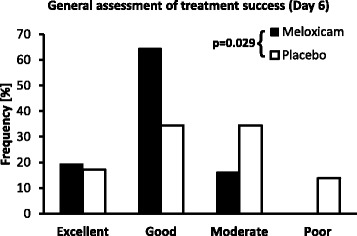
General assessment of treatment success on Day 6. Distribution (%) of the categories for the general assessment of analgesic and anti-inflammatory efficacy of treatment for the meloxicam and placebo treatment groups on Day 6. Treatment groups were significantly different based on chi-square analysis (p = 0.029)

**Figure 2:**
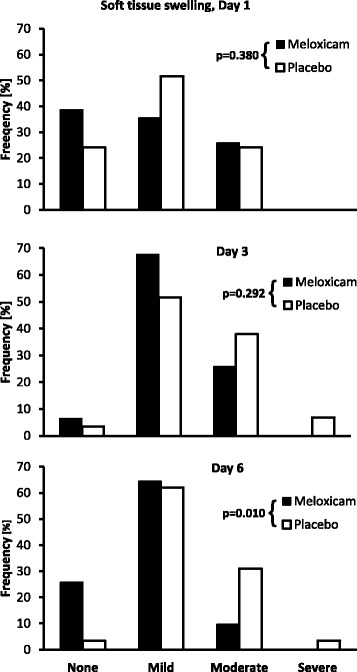
Effect on soft tissue swelling. Distribution (%) of the categories for soft tissue swelling at the surgical site on study days 1, 3, and 6 for the meloxicam and placebo treatment groups. Treatment groups were not significantly different on study days 1 and 3 (p = 0.380 and p = 0.292, respectively) but were significantly different on day 6 (p = 0.010) based on chi-square analysis with the "Moderate" and "Severe" categories combined

The significant treatment effect of meloxicam as seen for the primary efficacy variable LAMET was also reflected in secondary endpoints. The treatment groups were significantly different in terms of the general assessment of analgesic efficacy at the end of the study (p = 0.029, Figure 1). Based on combining the categories "Moderate" and "Poor" to reflect treatment failure and "Excellent" and "Good" to reflect treatment success, meloxicam treatment was judged to be successful in 83.9 % of cases, compared to 51.7 % in placebo-treated animals (p = 0.008). Soft tissue swelling at the surgical site did not differ between the two treatment groups on study days 1 and 3 (p = 0.380 and p = 0.292, respectively) but was significantly less in the meloxicam group on Day 6 (p = 0.010, Figure 2). In particular, the percent of horses categorized as having moderate to severe swelling of soft tissue at the surgical site on Day 6 was only 9.7 % in the meloxicam group compared to and 34.4 % in the placebo group.

The treatment group scores for local palpatory pain at the site of surgery, circumference of the most severely affected area of the affected limb, and the scores for wound healing were similar at the various assessment time points in the study. There was no evidence for any significant difference between the two treatment groups with respect to food intake, rectal temperature or heart rate at any of the assessment time points in the study. Post-surgery recovery assessments in terms of the number of attempts which the horses made to stand until they actually managed to remain standing upright and the interval between the time of extubation and the time when the horse was able to remain standing upright were similar for both groups. No abnormality was observed in the localisation of callus formation in more than 80 % of the cases in both treatment groups. No adverse events were observed in any of the horses, and all were returned to their owners at the end of the study.

## Discussion

Good pain control after surgery has been recognized as important component to prevent negative outcomes including poor wound healing and prolonged recovery [21]. Exacerbations of acute pain can lead to neural sensitization and release of mediators both peripherally and centrally, contributing to delayed recovery. This phenomenon, called wind up, was found to be caused by N-Methyl D-Aspartate (NMDA) receptor activation mediated central sensitization, long-term potentiation of pain (LTP), and transcription-dependent sensitization [22]. The knowledge of these pathways has led to increased awareness on post-operative pain and has changed the attitudes towards pain management [22]. Both in human medicine and in small animal medicine, post-operative intensive pain management is now standard [21,22].

In contrast, the utility of post-operative pain management in horses undergoing orthopaedic surgery is still a matter of controversial discussion, since pain is seen as a protective mechanism resulting in reduced usage of the affected limb [4]. The fallacy, however, that pain is protective and must be allowed to avoid risk for damage after surgery should be challenged. Post-operative analgesia is directed at aching pain, whereas sharp pain associated with inappropriate movements persists [21]. While theoretical evidence is strong in support of post-operative pain management in horses undergoing orthopaedic surgery, and while post-operative pain management is used at least in a fraction of horses undergoing castration [23], clinical proof of improved outcome due to pain management is limited. In fact, only one placebo-controlled small clinical field study in horsed was identified evaluating the use of NSAIDs in post-operative pain management [11]. In that study in horses undergoing arthroscopic surgery, phenylbutazone was found to reduce the pain severity if assessed using a composite pain score, but due to the low number of cases involved (15 on phenylbutazone and 10 on placebo), the study failed to demonstrate statistically significant and clinically meaningful effects. That study also demonstrated the difficulties associated with pain assessment in horses after surgery, which is especially challenging when multiple investigators are involved, resulting in inter-rater variability [24-26].

Therefore, the current study was designed to reduce variability where possible. The surgical condition selected, i.e. unilateral closed splint bone fracture with marked exostosis and development of connective tissue induration, is a condition found in sufficient frequency to recruit a sizable clinical study in a multi-centre setting. At the same time, the condition is relatively homogenous, and the surgical procedure involved is well defined and limited in extent, thereby reducing the variability within the trial population. Since no weight bearing structures are directly involved, horses can be expected not to suffer from unacceptable postoperative pain during rest, enabling the use of placebo. In fact, while the short-acting morphine derivative butorphanol was allowed as rescue medication, no case required its use. Instead of attempting to quantify pain, we selected lameness as the primary outcome in that it reflects the pain perception of the affected horse. Lameness quantification using the AAEP scale is a routine procedure well established at all participating study sites. Two different conditions were evaluated. LAMET was selected to represent the pain perception arising from tissue movements in the surgical region induced by forcing the horses to trot. LAMEW was selected to represent the pain perception during rest and walking, and this measure was selected to best reflect the overall suffering level during the recovery. As can be expected from the medical condition, LAMEW was very low throughout the study and did not differ between the placebo and the treatment group, indicating that placebo animals were not exposed to unacceptable pain (Table 4).

We selected meloxicam as the trial drug because it is licensed for use in horses for the alleviation of inflammation and relief of pain in both acute and chronic musculo-skeletal disorders, as well as for the relief of pain associated with colic [13]. Information on pharmacokinetic and pharmacodynamic relationship in orthopaedic conditions, as well as safety of single and multiple doses, is available enabling the selection of the dose and dosing interval [15,27,28]. While phenylbutazone is frequently used in horses, its use is associated with the highest rate of adverse events of NSAIDs, with a stronger effect on gastric mucosa as compared to meloxicam, and, therefore, was not considered as study medication [29,30].

Using this study design, we were able to demonstrate that preoperative and post-operative administration of meloxicam resulted in a significant reduction in lameness on the 6^th^ day after surgery, indicating that the recovery in animals receiving pain treatment was accelerated compared to placebo-treated animals. This positive effect was also reflected by the significantly improved overall clinical assessment of treatment success, reflecting both a significant better analgesia and a significant reduction in soft tissue swelling on day 6. The reduced lameness on day 6 is clinically relevant, since it reduces the risk for development of support-limb laminitis, an unfortunate sequela that can render prior surgery needless and that often leads to the demise of the affected horse [5].

The strongest clinical effects were seen on day 6 after surgery, while immediately after surgery there was no difference as assessed by number of attempts to stand until managing to remain standing and time from extubation to time to remaining to stand upright. This indicates that early recovery from surgery may rather be related to anaesthetic procedures, which did not differ between groups, than to post-operative pain control. In fact, the pain related to the selected surgical procedure can be expected to be rather limited, having little effect on time to recovery from anaesthesia. Our data indicate that, in conditions with limited surgical intervention, early recovery cannot be improved with post-operative pain management. On day 3 after surgery, there was a trend, although not statistically significant, towards reduced lameness (Table 3). The reduced efficacy on day 3 compared to day 6 was somewhat unexpected. One reason for the delayed manifestation of clinical benefit may be the selected treatment scheme. The analgesia of an intravenous dose of 0.5-1 mg/kg meloxicam lasts in horses up to 24 hours, supporting once daily dosing [15]. In our study, an intravenous dose of 0.6 mg/kg was given immediately prior to pre-medication of anaesthesia, and the first post-surgical oral dose was administered about 24 hours later in the morning on the day following the surgery. An analgesia gap due to switching from intravenous to oral dosing could be a potential concern for inappropriate post-surgical pain relief [4,27]. In future studies, a more aggressive early treatment, potentially involving a 2^nd^ intravenous dose, timed to bridge the gap until oral treatment reaches effective plasma levels, may result in even further improved outcome.

Our primary endpoint was based on lameness assessment using the 6-point AAEP scale for lameness at trot. Unfortunately, lameness evaluation is afflicted with low reproducibility, especially if low grades of lameness are involved, even if experienced raters are involved [18]. When the mean lameness AAEP score was lower than 1.5, the inter-rater agreement whether a respective limb was lame or not was only 61.9 %, while it was 93.1 % if the lameness score was >1.5 [18]. In view of this problem, we evaluated whether a reduction of the number of lameness scores could increase the sensitivity of this scale to treatment effects. For this purpose, the AAEP scores 0 and 1 were grouped to present the positive outcome of none or minimal lameness, while the scores 3–5 were grouped as moderate to severe lameness, leaving score 2 as well recognizable, but mild lameness. This lameness scale, revised to represent clinically relevant differences, aimed at reducing the uncertainty of evaluating low grades of lameness and also at avoiding the need for rating different grades of moderate to severe lameness. Based on application of this scale, the study result obtained was essentially similar but the difference on day 3 became almost statistically significant (p-value = 0.053, Table 3). This indicates that the reduced lameness scale may present a method which is less affected by inter-rater variability.

In carefully reviewing the data, it became evident that, despite the blinded randomization of the cases resulting in equal distribution of cases with regard to race, sex, age, weight, and lameness duration, there was a slight but statistically non-significant difference in pre-randomization lameness grade, with a tendency towards less severe lameness in the meloxicam group. While the study aimed at evaluating the drug effect on surgical pain, this slight difference could have influenced the study outcome. Therefore, the OLR analysis of lameness was repeated for day 3 and 6 with adjustment for day 0 values. This adjustment was without effect on the overall study outcome on day 6.

During the course of the study, no treatment-related adverse events were recorded, but this was expected given that meloxicam was dosed in accordance with the label and that known NSAID related toxicity, including gastric ulceration and necrosis, require longer treatment duration to develop [29,31]. In addition, NSAIDs have not been found to have a detrimental effect on wound healing [32]. In fact, we showed in this study that the scores for wound healing were similar at the various assessment time points in the study, but the treatment resulted in significantly reduced swelling of soft tissue on day 6.

## Conclusions

We have demonstrated for the first time that pre and post-operative meloxicam administration has a statistically significant beneficial effect on recovery in horses undergoing orthopaedic surgery of limited degree. This positive effect could be identified and verified in a relatively small placebo-controlled field study population of 60 evaluable horses by selecting a homogenous study population and utilizing a subjective outcome measure, i.e. lameness at trot. The utilization of a clinically relevant lameness scale increased the sensitivity of the study to treatment effects. Our results for the first time support the empirical conclusion drawn by experienced clinicians that post-operative pain management using clinically safe NSAIDs, such as meloxicam, is advantageous in equine orthopaedic surgery.

## Endnotes

^a^Metacam® 20 mg/ml Solution for Injection, Boehringer Ingelheim Vetmedica GmbH, Ingelheim, Germany.

^b^Metacam® 15 mg/ml Oral Suspension for Horses, Boehringer Ingelheim Vetmedica GmbH, Ingelheim, Germany.

## Abbreviations

AAEP: American Association of Equine Practitioners
GCP: Good clinical practice
CRLAT: Clinically relevant lameness at trot
LAMET: Lameness at trot
LAMEW: Lameness at walk
NSAID: Non-steroidal anti-inflammatory drug
